# CO_2_ emissions from C40 cities: citywide emission inventories and comparisons with global gridded emission datasets

**DOI:** 10.1088/1748-9326/acbb91

**Published:** 2023-02-28

**Authors:** D Y Ahn, D L Goldberg, Toby Coombes, Gary Kleiman, S C Anenberg

**Affiliations:** 1 Milken School of Public Health, George Washington University, Washington, DC, United States of America; 2 C40 Cities Climate Leadership Group Inc., New York, NY, United States of America; 3 Orbis Air, LLC, Concord, MA, United States of America

**Keywords:** EDGAR, ODIAC, C40 cities, GPC inventory, CO_2_, emissions, emission trends

## Abstract

Under the leadership of the C40 Cities Climate Leadership Group (C40), approximately 1100 global cities have signed to reach net-zero emissions by 2050. Accurate greenhouse gas emission calculations at the city-scale have become critical. This study forms a bridge between the two emission calculation methods: (a) the city-scale accounting used by C40 cities—the Global Protocol for Community-Scale Greenhouse Gas Emission Inventories (GPC) and (b) the global-scale gridded datasets used by the research community—the Emission Database for Global Atmospheric Research (EDGAR) and Open‐Source Data Inventory for Anthropogenic CO_2_ (ODIAC). For the emission magnitudes of 78 C40 cities, we find good correlations between the GPC and EDGAR (*R* ^2^ = 0.80) and the GPC and ODIAC (*R* ^2^ = 0.72). Regionally, African cities show the largest variability in the three emission estimates. For the emission trends, the standard deviation of the differences is ±4.7% yr^−1^ for EDGAR vs. GPC and is ±3.9% yr^−1^ for ODIAC vs. GPC: a factor of ∼2 larger than the trends that many C40 cities pledged (net-zero by 2050 from 2010, or −2.5% yr^−1^). To examine the source of discrepancies in the emission datasets, we assess the impact of spatial resolutions of EDGAR (0.1°) and ODIAC (1 km) on estimating varying-sized cities’ emissions. Our analysis shows that the coarser resolution of EDGAR can artificially decrease emissions by 13% for cities smaller than 1000 km^2^. We find that data quality of emission factors (EFs) used in GPC inventories vary regionally: the highest quality for European and North American and the lowest for African and Latin American cities. Our study indicates that the following items should be prioritized to reduce the discrepancies between the two emission calculation methods: (a) implementing local-specific/up-to-date EFs in GPC inventories, (b) keeping the global power plant database current, and (c) incorporating satellite-derived CO_2_ datasets (i.e. NASA OCO-3).

## 1. Introduction

Cities currently account for about 70% of anthropogenic greenhouse gas (GHG) emissions (UN-Habitat, 2019). Urban emissions could increase as the global population percentage living in cities is expected to increase from 55% in 2018 to 68% in 2050 (U.N. 2019). Given the significance of urban GHG emissions, many cities have signed pledges to reduce GHG emissions as a contribution to mitigating the worst effects of climate change. The C40 Cities Climate Leadership Group (hereafter, C40) is a network of mayors collaborating to reduce urban GHG emissions and influence the global climate agenda. As of 2021, C40 comprises 97 cities, which account for a population of 582 million and 20% of the global Gross Domestic Product (C40 2021). Under the leadership of C40 and six international organizations, 1122 cities have signed onto the Race to Zero campaign, which aims to halve GHG emissions by 2030 and reach net zero emissions by 2050 (UNFCCC 2022).

The Global Protocol for Community-Scale Greenhouse Gas Emission Inventories (GPC) is a guideline tool for cities to estimate annual GHG emissions (C40, ICLEI, and WRI 2014). The GPC provides the principles and rules for calculating emissions consistent with IPCC Guidelines, but it does not require specific methodologies. Cities collect activity data and emission factors (EFs) based on availability and consistency with their country’s national reported emissions. Since 2014, C40 cities have reported GPC-based GHG emission inventories to the Carbon Disclosure Project (CDP). The consistency and transparency of GPC inventories enable cross-city emissions comparisons, emission mitigation policy assessments, and consumption-based carbon footprint estimations (Lopes Toledo and Lèbre La Rovere 2018, Nangini *et al* 2019, Salvia *et al* 2021, Wiedmann *et al* 2021, Kongboon *et al* 2022).

The emissions research community has developed gridded CO_2_ emissions datasets at various scales, including urban (Meng *et al* 2014, Zhu *et al* 2019, Gurney *et al* 2019c, Roest *et al* 2020, Moran *et al* 2022), regional (Kurokawa *et al* 2013, Gately and Hutyra 2018), national (Cai *et al* 2018, Bun *et al* 2019, Gurney *et al* 2019a), and global scales (Wang *et al* 2013, Asefi-Najafabady *et al* 2014, Janssens-Maenhout *et al* 2017, McDuffie *et al* 2020, Oda and Maksyutov 2020, Jones *et al* 2021). The Emission Database for Global Atmospheric Research (EDGAR) (Janssens-Maenhout *et al* 2017) and the Open‐Source Data Inventory for Anthropogenic CO_2_ (ODIAC) (Oda *et al* 2018) are well-established emissions datasets with frequent updates covering up to recent years globally. The EDGAR and ODIAC’s global coverage and frequent updates enable estimating CO_2_ emissions from global cities, such as the C40 network. EDGAR and ODIAC have been used to estimate urban emissions for serving as the first guess for atmospheric inversion studies or directly for policy-relevant purposes (Oda *et al* 2017, Crippa *et al* 2019, 2021, Lauvaux *et al* 2020). Correspondingly, assessing the accuracy of global gridded emissions at sub-national scales has become critical (Hogue *et al* 2016, Gately and Hutyra 2017, Gurney *et al* 2019b, Chen *et al* 2020).

Comparisons of the gridded emission datasets and local inventories have revealed uncertainties and biases associated with individual activity data, EFs, and downscaling methods (Gately and Hutyra 2017, Hutchins *et al* 2017, Wang and Cai 2017, Nangini *et al* 2019, Oda *et al* 2019, Gurney *et al* 2019b, 2021, Ahn *et al* 2020, Chen *et al* 2020, Roest *et al* 2020, Han *et al* 2020b, 2020c, Moran *et al* 2022). Chen *et al* (2020) compared ODIAC’s estimate of CO_2_ emissions for 14 global cities to their emission estimates retrieved from literature—the earliest, median, and latest accounting years for the 14 global cities were 2000, 2005, and 2011 (Kennedy *et al* 2009, 2010). Chen *et al* (2020) found large variability between the ODIAC and emission inventory estimates, ranging from −62% (ODIAC < Inventory) for Manhattan, USA, to +148% for Sao Paulo, Brazil.

We assess the level of agreement between city-level emission accounting (GPC inventories) and global gridded emission datasets (EDGAR and ODIAC) in estimating citywide CO_2_ emissions and their trends. We choose the two gridded datasets—EDGAR and ODIAC—given their global coverage over a long-term period (1970–2018 for EDGARv6.0 and 2000–2019 for ODIAC 2020b). We expand on the previous literature by Chen *et al* (2020) by (a) including more cities for a total of 78 globally, (b) updating the most recent emission inventory reported (2014–2019), (c) using GPC inventories as opposed to using emission estimates from the literature, (d) including both EDGAR and ODIAC’s emission estimates, and (e) comparing emission trends. Further, we investigate the source of discrepancies between GPC inventories and gridded emission datasets. The results presented in this study can inform city staff in charge of local emission inventory and developers of gridded emission datasets about the current level of agreement for urban CO_2_ emission estimates on the global scale and provide potential opportunities for improvements in the emission estimating methods.

## 2. Materials and methods

### 2.1. CO_2_ emissions data: GPC inventory, EDGAR, and ODIAC

The C40 GHG Dashboard compiles the GPC GHG emission inventories reported by 82 C40 cities (46 countries) to CDP. The emission accounting years for these inventories vary by city, ranging from 1990 to 2020. The C40 GHG Dashboard data are obtained from the C40 knowledge hub website (www.c40knowledgehub.org/s/article/C40-cities-greenhouse-gas-emissions-interactive-dashboard) (C40 2022). We isolate fossil fuel CO_2_ (FFCO_2_) emissions by applying a scaling factor of 0.982 to the total fossil-fuel GHG emissions from the Dashboard data (figure S1). See text S1 for detailed methods for isolating FFCO_2_ emissions from each CO_2_ emissions dataset.

EDGAR’s annual fossil CO_2_ emissions estimates are primarily based upon energy data from the International Energy Agency and national-specific EFs (i.e. EPA AP-42, EMEP/EEA, and IPCC). These annual CO_2_ emissions by sub-sectors are spatially distributed into 0.1° grids globally using ∼300 spatial proxies (i.e. population/urban settlements (JRC GHSL), point source distributions (CARMA, EPRTR), and line sources (OpenStreetMap) (Janssens-Maenhout *et al* 2017, Crippa *et al* 2021). Annual sector-specific CO_2_ emissions from EDGARv6.0 for fossil CO_2_ sources (‘CO2_excl_short-cycle_org_C’), available for the 1970–2018 period, are obtained from the JRC EDGAR website (https://edgar.jrc.ec.europa.eu/dataset_ghg60).

ODIAC downscales national fossil CO_2_ emission estimates from the Carbon Dioxide Information Analysis Center (CDIAC, (Marland and Rotty 1984)) into 1 km grids globally (Oda and Maksyutov 2011, Oda *et al* 2018). The CDIAC estimates national fossil CO_2_ emissions using energy statistics published by the United Nations. For spatial disaggregation, ODIAC uses the Defense Meteorological Satellite Program (DMSP, (Elvidge *et al* 1999)) nightlight imagery as a spatial proxy for non-point sources and the carbon monitoring action (CARMA, (Wheeler and Ummel 2008)) data to locate power plant emissions. ODIAC version 2020b (ODIAC2020b), available for 2000–2019, is obtained from the Center for Global Environmental Research, National Institute for Environmental Studies (NIES) website (https://db.cger.nies.go.jp/dataset/ODIAC/).

### 2.2. Aligning CO_2_ emissions from GPC inventory, EDGAR, and ODIAC for comparison

We align the three datasets—GPC inventory, EDGAR, and ODIAC—by sector, time, and spatial area to enable comparisons between them. For the sectoral alignment, we include scope 1 FFCO_2_ emissions from the five sectors—residential, commercial, industrial, energy, and transportation—from the GPC inventory, EDGAR, and ODIAC, respectively. Four C40 cities—Bangkok, Dakar, Hong Kong, and Phoenix—are excluded due to missing emissions data for one or more subsectors in the GPC inventories. See text S1 for detailed methods for isolating FFCO_2_ emissions from each CO_2_ emissions dataset.

For the temporal alignment of annual emission estimates, we choose the most recent accounting year with estimates from all three emissions datasets available for each city. Of the 82 C40 cities with self-reported inventories, 74 cities have inventories for years between 2014 and 2018, which align with the years available from EDGAR version 6.0 and ODIAC version 2020b. Four cities—Rotterdam, Bengaluru, Mumbai, and Washington, DC—have their first available GPC inventory for 2019, and we use EDGAR emissions for 2018 for the comparison. For the temporal alignment of emission trend estimates, we use the earliest and latest accounting years available before 2019 for each city. Of the 82 C40 cities, 39 cities have submitted at least two complete inventories between 2000 and 2018.

**Figure 1.**
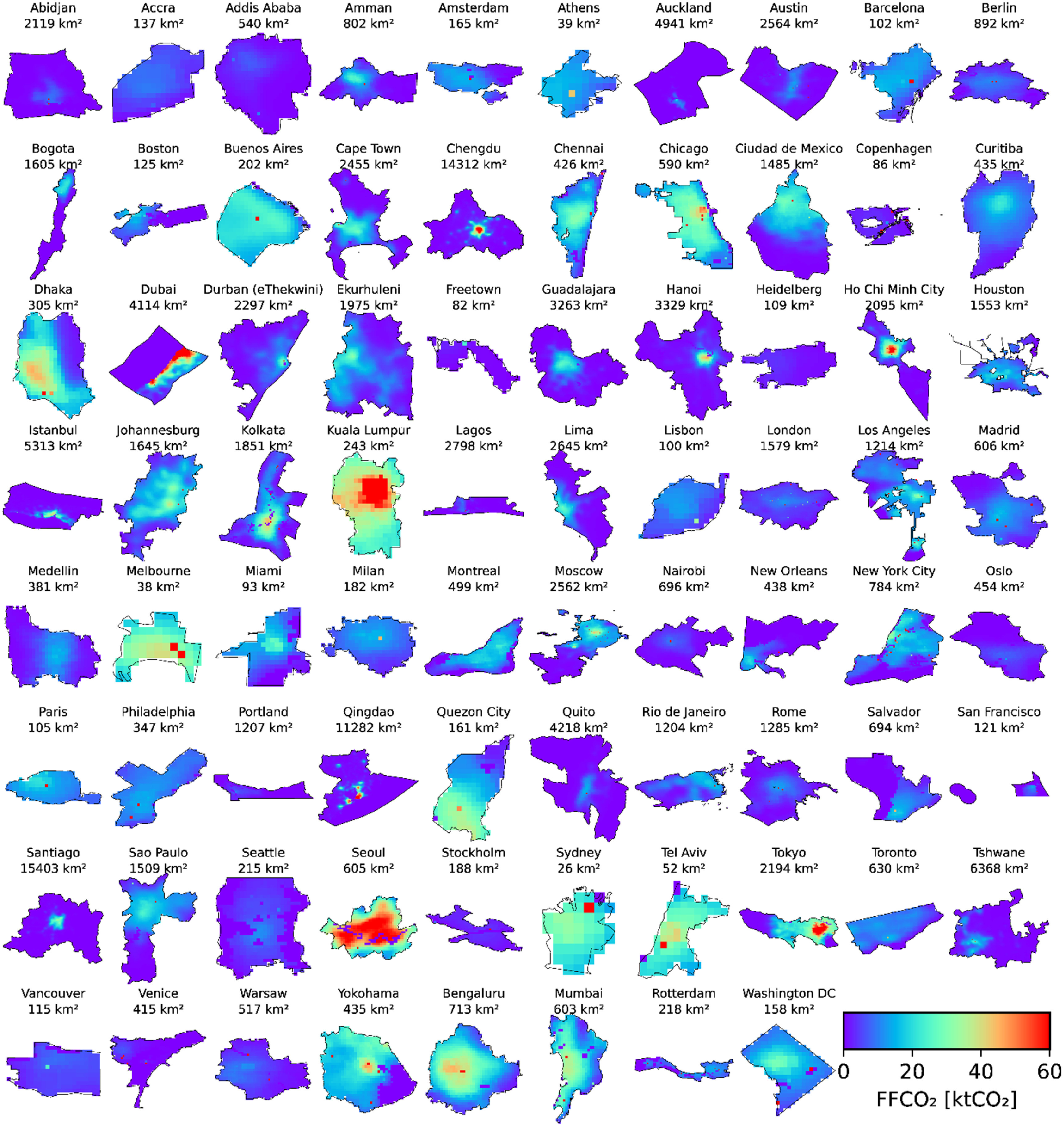
Overview of the 78 C40 cities analyzed in this study. The area size for each city is shown. Color scales show ODIAC’s fossil fuel CO_2_ emissions (unit: ktCO_2_ km^−2^ yr^−1^, year 2015).

The types of geographical boundaries used to construct the GPC inventories are the administrative boundary of a local municipality (*N* = 55), broader metropolitan area (*N* = 14), province/district/state/county (*N* = 6), and others (*N* = 3, i.e. Comprehensive Land Use Plan). For the spatial alignments, we aggregate CO_2_ emissions from EDGAR and ODIAC by sampling grids within the same geographical boundary used to construct the GPC inventory of each city (figure 1). For EDGAR grid cells (0.1°) overlapping the city boundary line, we scale emissions by the ratio of the grid area within the city boundary to the grid area of the 0.1° grid cell. For ODIAC grid cells (1 km), we aggregate emissions of the grid cells whose center coordinates are within the boundary and do not attempt to scale emissions.

## 3. Results

### 3.1. Scope 1 FFCO_2_ emissions of 78 C40 cities

#### 3.1.1. GPC GHG emissions inventory

The GPC guideline requires cities to report total GHG emissions attributable to activities within the city’s geographic boundary, termed the BASIC level. The BASIC level covers scope 1 (i.e. emissions from sources within the city) and scope 2 (i.e. emissions occurring due to the use of grid-supplied energy) emissions from stationary energy and transportation sectors, and scope 1 and scope 3 (i.e. emissions occurring outside the city due to activities within the city) emissions from the waste sector (Fong *et al* 2014). See figure 2 of Fong *et al* (2014) for the complete list of subsectors covered by BASIC level. It is important to note that the BASIC level covers emissions from grid-supplied energy consumption (scope 2) while excluding the emissions from energy generation supplied to the grid (scope 1).

**Figure 2.**
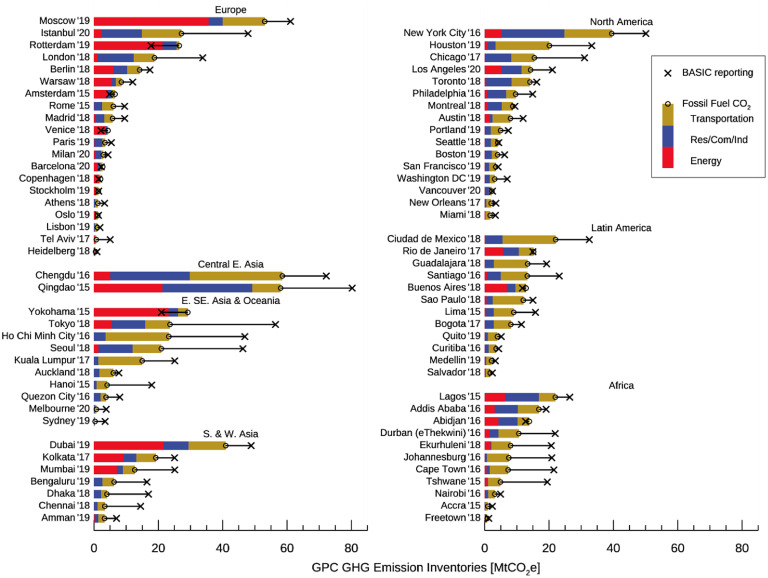
The total greenhouse gas emissions (termed ‘BASIC’ in GPC, symbol X) and scope 1 fossil fuel CO_2_ (FFCO_2_, symbol O) emissions in the GPC inventories submitted by 78 C40 cities. Each Inventory’s accounting year is shown next to the city name (i.e. ′19 for 2019). Per-capita and per-area FFCO_2_ emissions for the same cities are shown in figures 3 and S2. The percentage sectoral compositions of FFCO_2_ emissions are shown in figure S3.

On average, the percentage of FFCO_2_ in total GHG emissions (‘BASIC’) is 57% for Africa, 77% for Central East Asia, 51% for South and West Asia, 84% for Europe, 76% for Latin America, 70% for North America, and 49% for South and West Asia cities, with the global mean of 69%. For the nine cities—Abidjan, Amsterdam, Barcelona, Buenos Aires, Copenhagen, Rio de Janeiro, Rotterdam, Venice, and Yokohama—the BASIC emissions are smaller than the FFCO_2_ emissions as these cities are net exporters of electricity toward surrounding areas.

Based on GPC inventories, 890 million tons (MtCO_2_) of FFCO_2_ are emitted from 78 C40 cities annually (figure 2). This magnitude of emissions is larger than Germany’s annual FFCO_2_ emissions (703 MtCO_2_, 6th largest emitting country) (Crippa *et al* 2020). Across the 78 C40 cities, the energy sector contributes 17% (1*σ*: ±23%) to citywide FFCO_2_ emissions, the residential/commercial/industrial sector contributes 32% (1*σ*: ±17%), and the transportation sector contributes 51% (1*σ*: ±22%). Regionally, cities in Europe have the largest energy sector contribution to CO_2_ emissions in this subset of cities (33 ± 30%), and cities in North America have the lowest energy sector contribution (7 ± 10%). Transportation contributes the most to FFCO_2_ emissions in cities in Latin America (65 ± 18%) and the least in Central East Asian cities (32 ± 24%).

**Figure 3.**
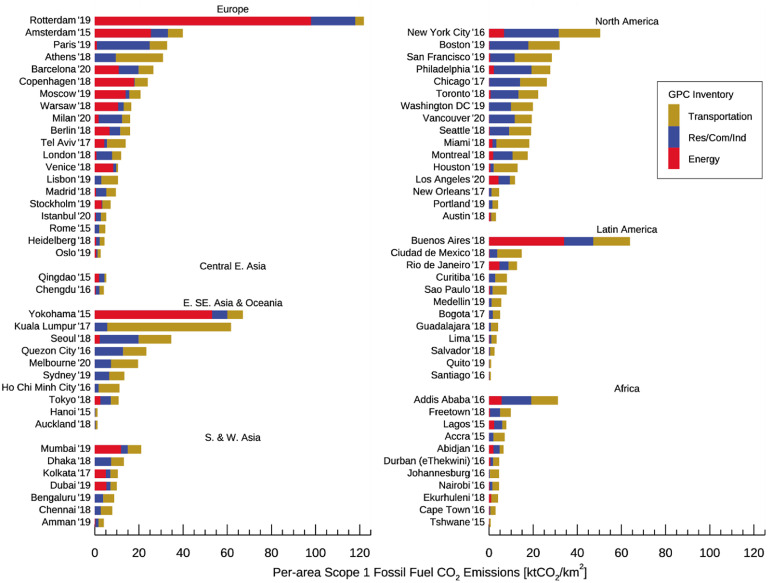
Per-area scope 1 fossil fuel CO_2_ (FFCO_2_) emissions in the GPC-based inventories submitted by 78 C40 cities. Each inventory’s accounting year is shown next to the city name. The types of boundaries used to construct GPC inventories are the administrative boundary of a local municipality (*N* = 55), broader metropolitan area (*N* = 14), province/district/state/county (*N* = 6), and others (*N* = 3, i.e. Comprehensive Land Use Plan). See figure S2 for per-capita FFCO_2_ emissions for 78 cities.

Socioeconomic factors such as population, area, and population density show scaling relationships with citywide CO_2_ emissions (Ribeiro *et al* 2019). According to GPC inventories of 78 global cities, citywide FFCO_2_ emissions show large variability, ranging from 0.4 MtCO_2_ for the city of Sydney, Australia, to 59 MtCO_2_ for Chengdu, China. Such variability is mainly due to the difference in population and land area within each city boundary. Population (and land area) is 14.7 million (14 312 km^2^) for Chengdu and 0.25 million (26 km^2^) for the city of Sydney (Note: the Sydney metropolis’ covers 5.2 million people in 12 368 km^2^). Figure 3 shows that the per-area FFCO_2_ emission for Sydney is 13.4 MtCO_2_ km^−2^ (citywide 0.3 MtCO_2_), greater than Tokyo’s per-area emissions of 10.8 MtCO_2_ km^−2^ (citywide: 23.6 MtCO_2_), highlighting the importance of using the matching geographical boundary for the emission comparison study. Cities with power plants within their geographical boundary tend to have larger scope 1 FFCO_2_ emissions (i.e. Rotterdam). In this study, we do not consider emissions occurring due to the import/export of electricity.

#### 3.1.2. GPC inventory, EDGAR, and ODIAC

**Figure 4.**
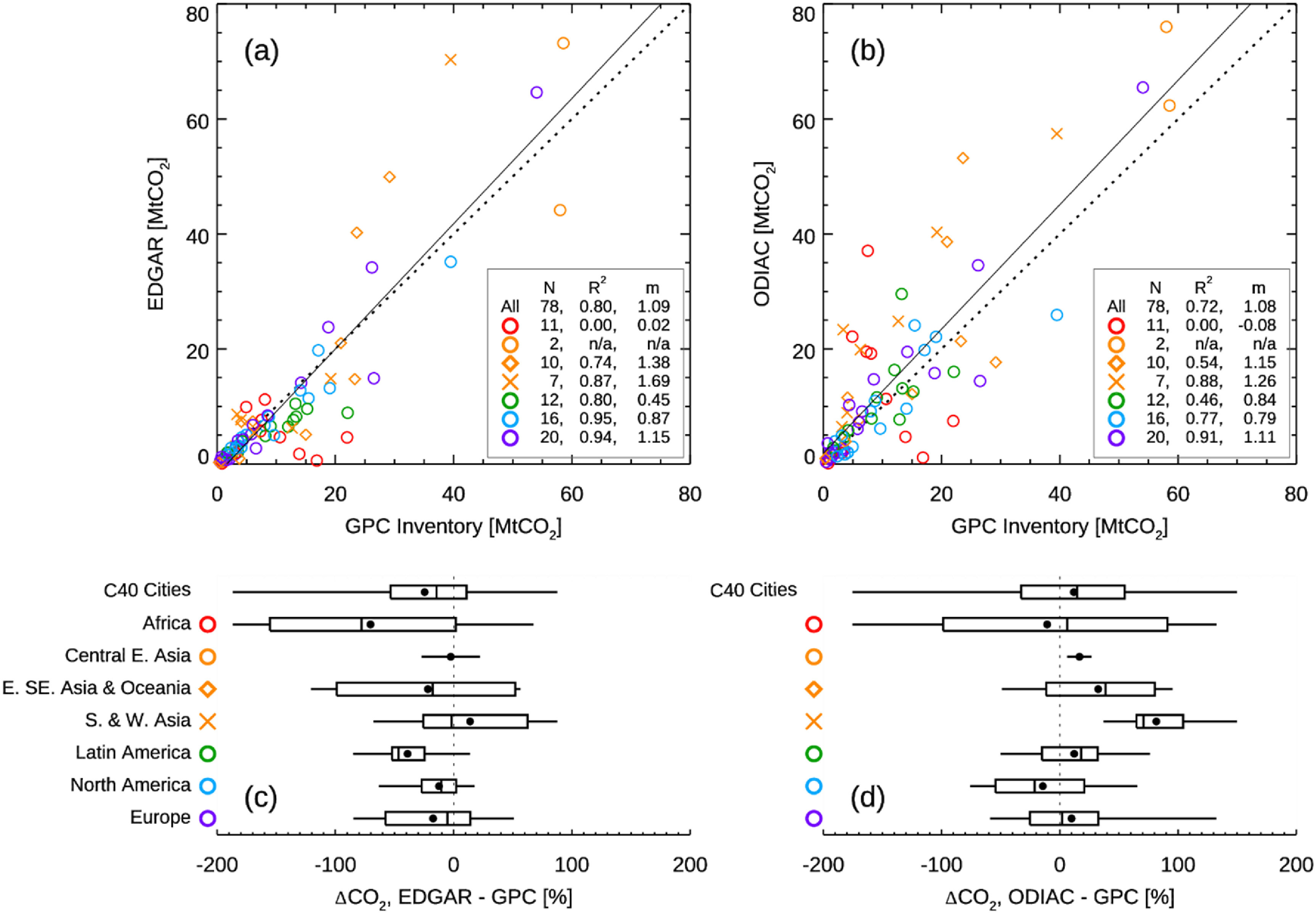
Comparison of scope 1 fossil fuel CO_2_ (FFCO_2_) emissions for 78 C40 cities estimated from the GPC emission Inventories against the EDGAR (left panels) and ODIAC (right panels). Different colors and symbols indicate the regions that cities belong to, as labeled in (b). The dotted and solid lines indicate the 1-to-1 ratio and the ordinary least squares fit, respectively. Bottom panels show the percentage difference of the emission estimates (ΔCO_2_ = (Gridded Dataset − GPC)/mean(Gridded Dataset, GPC) × 100). The box and whisker plot indicates the minimum, lower quartile, median, upper quartile, and maximum of the percentage differences. The black circle symbol indicates the mean percentage differences. See figure S5 for the direct comparison between EDGAR and ODIAC.

Figure 4 shows FFCO_2_ emissions in GPC inventories for the 78 C40 cities compared to emission estimates from the two global gridded emission datasets: EDGAR and ODIAC. Accounting years of the GPC inventories chosen for comparison range from 2014 to 2019 (median year: 2018). EDGAR’s estimates of FFCO_2_ emissions show a good correlation with GPC inventory estimates, with an *R* ^2^ value of 0.80 and the slope of ordinary least squares (OLS) fit as 1.09. Regionally, cities in North America show the highest *R* ^2^ value of 0.95, followed by cities in Europe (*R* ^2^ = 0.94). Across 78 cities, the mean and standard deviation of the relative difference between EDGAR and GPC inventory estimates are −25 ± 53% (Relative difference = (EDGAR − GPC)/mean(EDGAR, GPC) × 100). African cities show the largest relative difference (−70 ± 84%). For Latin American cities, EDGAR emissions tend to be consistently smaller than GPC inventory emissions: 11 of 12 Latin American cities show EDGAR emissions lower than GPC inventory emissions. Only Salvador, Brazil, shows EDGAR emissions greater than GPC emissions by 13%.

ODIAC shows a good correlation with GPC inventory estimates for the 78 C40 cities, with an *R* ^2^ of 0.72 and the slope of OLS fit as 1.08. Regionally, European cities show the highest *R* ^2^ of 0.91. Across all 78 cities, the mean and standard deviation of the relative difference between ODIAC and GPC inventory estimates are 12 ± 62%. For six cities in South and West Asia, ODIAC emission estimates are consistently larger than the GPC inventory emission estimates (RD = 82 ± 36%). African cities show a relative difference of −11 ± 111%, the largest variability across all regions.

### 3.2. Trends in scope 1 FFCO_2_ emissions for C40 cities

#### 3.2.1. GPC emissions inventory

**Figure 5.**
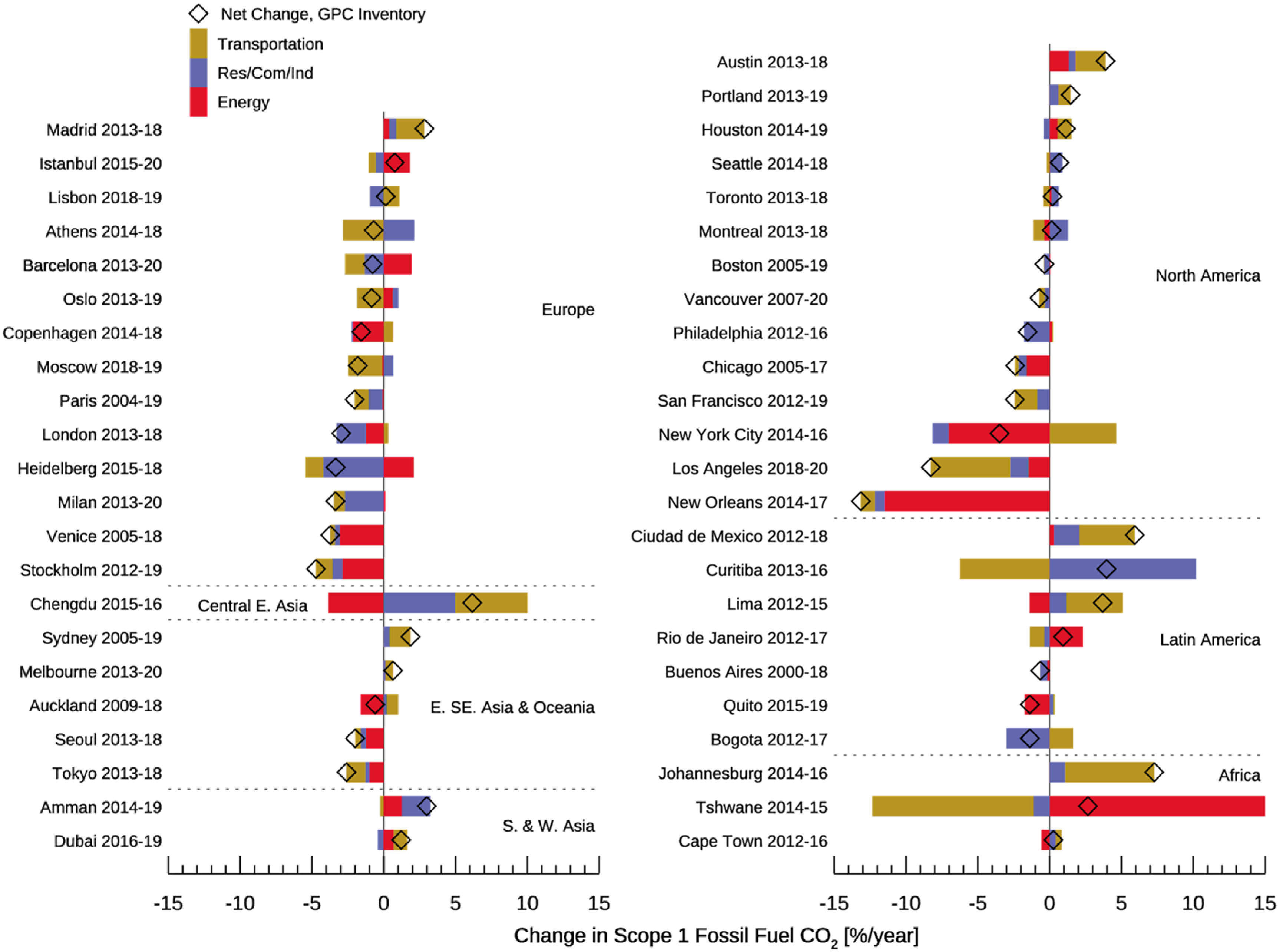
Percentage changes in FFCO_2_ emissions between the earliest and the latest accounting years of the GPC inventories for 46 C40 cities (Change = (CO_2,Latest_ − CO_2,Earliest_)/CO_2,Earliest_/Δyears × 100, unit: % yr^−1^). Two accounting years are shown next to city names. The colored bars indicate the sectoral emission changes, and the diamond symbol indicates the net emission change. Thirty-two cities in figure 3 are excluded as they only have one year of available data or missing data in the time series. Figure S4 shows the total percentage changes in emissions between the two accounting years.

Figure 5 shows the percentage change rates of FFCO_2_ emissions between the earliest and latest accounting years for which GPC emissions inventories are available. For the 46 C40 cities with more than one accounting year, the temporal gaps between the two accounting years range from 1 to 18 years (median: 5 years). Of the 46 C40 cities, 25 cities show declines in FFCO_2_ emissions over time. New Orleans, USA, shows the fastest decline rate of −13.1% yr^−1^ between 2005 and 2018. The rapid decline of CO_2_ emissions in New Orleans results from the 2016 closure of the Michoud power plant (EPA 2022a). The median emissions decline rate is −2.0% yr^−1^ for the 25 cities. The other 21 cities have shown increased emissions since their earliest accounting years, ranging from +0.1% yr^−1^ to +7.3% yr^−1^ (median: +1.5% yr^−1^). The transportation sector emissions have declined for 26 out of 46 cities since the first accounting years. For the residential/commercial/industrial sector, 26 out of 46 cities show declines in emissions. Energy sector emissions have declined in 19 cities and increased in another 19, while the remaining 8 cities have no in-city energy sector CO_2_ emissions.

#### 3.2.2. Comparison between GPC inventory and gridded datasets

**Figure 6.**
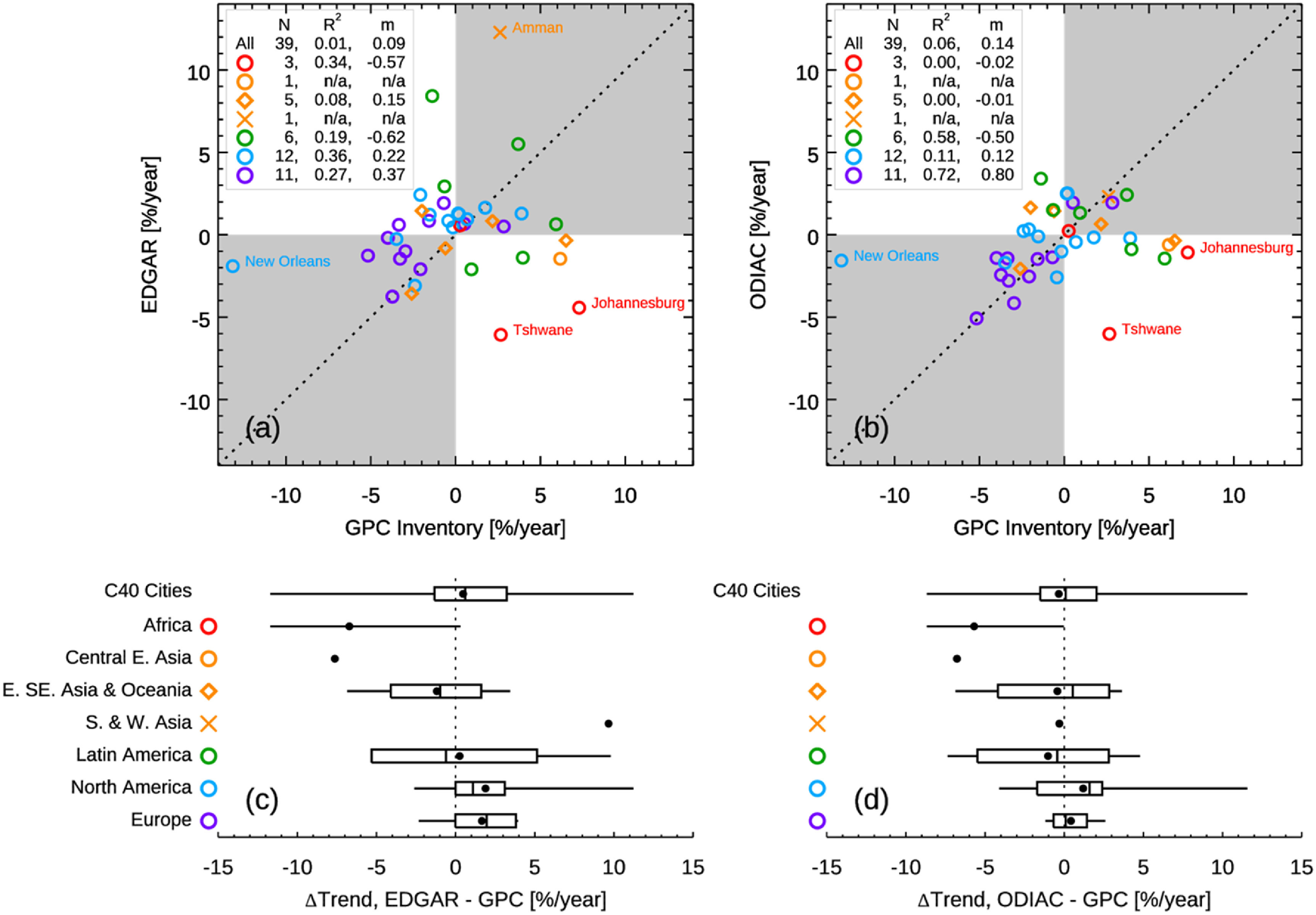
The upper panels show FFCO_2_ emissions trends estimated from the GPC inventory, EDGAR (a), and ODIAC (c). Different colors and shapes of symbols indicate C40 regions that cities belong to, as labeled in (b). The lower panels show differences in the trend estimates between EDGAR and GPC (b) and ODIAC and GPC (d) (ΔTrend = Trend_Gridded Dataset_ − Trend_GPC_). The box and whisker plot indicates the minimum, lower quartile, median, upper quartile, and maximum differences. The black circle symbol indicates the mean differences. See figure S5 for the direct comparison between EDGAR and ODIAC.

Figure 6 compares the trends in FFCO_2_ emissions for 39 C40 cities, estimated from the GPC inventory, EDGAR, and ODIAC. For the comparison analysis, we calculate the trends in emissions using the emissions for two accounting years as followings: (Trend = (CO_2,Latest year before 2019_ − CO_2,Earliest year_)/CO_2,Earliest_/Δyears × 100, unit: % yr^−1^). The comparison analysis excludes seven cities listed in figure 5—Dubai, Houston, Istanbul, Lisbon, Los Angeles, Moscow, and Quito—as they have less than two GPC inventories before 2019. Out of 39 cities in the comparison, the number of cities that show the same directional changes in emissions is 23 for GPC vs. EDGAR and 24 for GPC vs. ODIAC (Quantiles I and III in figures 6(a) and (b)). The mean and standard deviation of differences in trend estimates (Trend_Gridded Dataset_ − Trend_GPC_) is 0.5 ± 4.7% yr^−1^ for EDGAR vs. GPC and −0.3 ± 3.9% yr^−1^ for ODIAC vs. GPC.

Two African cities—Johannesburg and Tshwane, South Africa—show the largest discrepancies in the trend estimates, having opposite directional changes. For Latin American cities, the trend estimates showed overall good agreement with a mean difference of 0.3% yr^−1^ for EDGAR vs. GPC and −1.0% yr^−1^ for ODIAC vs. GPC. However, the trend estimates for Latin American cities showed significant variability: the standard deviation of the difference is ±6.0% yr^−1^ for EDGAR vs. GPC and ±4.5% yr^−1^ for ODIAC vs. GPC, both values being the second largest following African cities (figures 6(b) and (d)). European cities showed the best agreement in the trend estimates, having a trend difference of 1.7 ± 2.0% yr^−1^ for EDGAR vs. GPC and 0.4 ± 1.2% yr^−1^ for ODIAC vs. GPC, followed by North American cities and E. SE Asian & Oceanic cities. The city of New Orleans, USA, shows a noticeably larger difference than other North American cities (the GPC inventory: −13.1% yr^−1^, EDGAR: −1.9% yr^−1^, ODIAC: −1.6% yr^−1^).

### 3.3. Sources of discrepancies between GPC inventory, EDGAR, and ODIAC

#### 3.3.1. Spatial resolutions of ODIAC (1 km) and EDGAR (0.1°)

**Figure 7.**
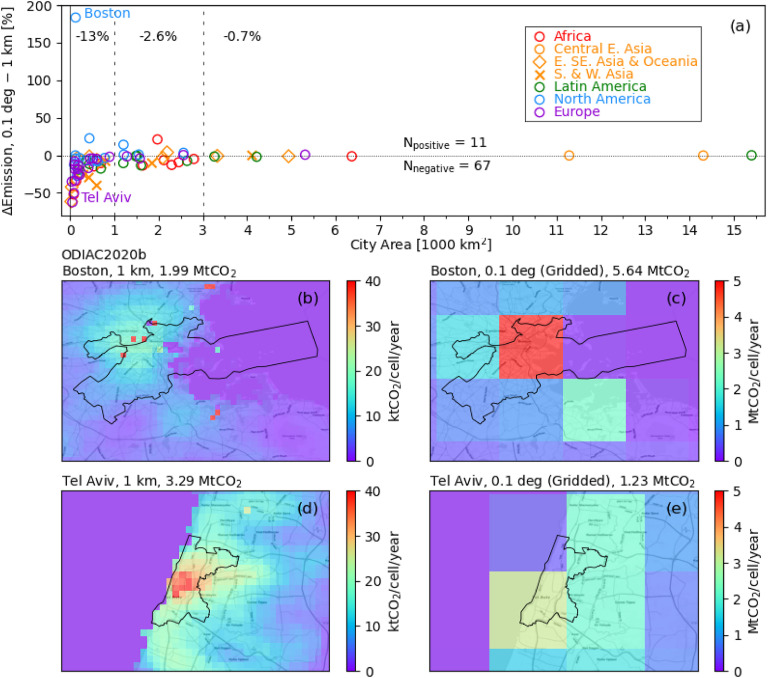
Sensitivity analysis of citywide CO_2_ emission estimates from ODIAC in two spatial resolutions: 1 km (original, (b) and (d)) and 0.1° (gridded (c) and (e)). To estimate the citywide emissions from 0.1° cells, we use the spatial area scaling approach as described in section 2.2. The top panel (a) shows the percentage differences in emission estimates from 0.1° and 1 km ODIAC for 78 C40 cities. The panel indicates the mean percentage differences for three area ranges—smaller than 1000 km^2^, between 1000 km^2^ and 3000 km^2^, and greater than 3000 km^2^. The number of cities with negative and positive differences are shown (*N*_negative_, *N*_positive_). The lower panels show the maps of two cities with the highest (Boston, USA (b) and (c)) and the lowest percentage differences (Tel Aviv, Israel (d) and (e)). See section 4.2 for the discussion on the impact of spatial resolutions of a gridded emission product on estimating citywide emission estimates.

We investigate the impact of spatial resolutions of a gridded product—1 km vs. 0.1°—on estimating citywide CO_2_ emissions as follows. First, ODIAC’s 1 km grid cells are spatially aggregated into 0.1° grid cells. Then, citywide FFCO_2_ emissions for 78 C40 cities are estimated from 0.1° gridded ODIAC using the same area scaling approach used for EDGAR (see section 2.2). Figure 7(a) shows the percentage differences of citywide CO_2_ emissions estimated from 0.1° gridded and the original 1 km ODIAC. We find that gridding of 1 km pixels into 0.1° pixels decreased emission estimates for 67 out of 78 C40 cities, while 11 cities showed an increase in emission estimates. The magnitude of decrease or increase in emission estimates tends to be smaller for larger area cities: the mean percentage differences in emission estimates between the 0.1° and 1 km ODIAC is −13% for the cities smaller than 1000 km^2^ (*N* = 47), −2.6% for cities between 1000 km^2^ and 3000 km^2^ (*N* = 21), and −0.7% for cities larger than 3000 km^2^ (*N* = 10). Boston, USA (figures 7(b) and (c)) and Tel Aviv, Israel (figures 7(d) and (e)) show the largest (+183%) and smallest (−63%) percentage differences, respectively. We discuss the contributing factors to the varying impacts of spatial resolutions and their policy implications in section 4.2.

#### 3.3.2. Spatial disaggregation errors in EDGAR and ODIAC

**Figure 8.**
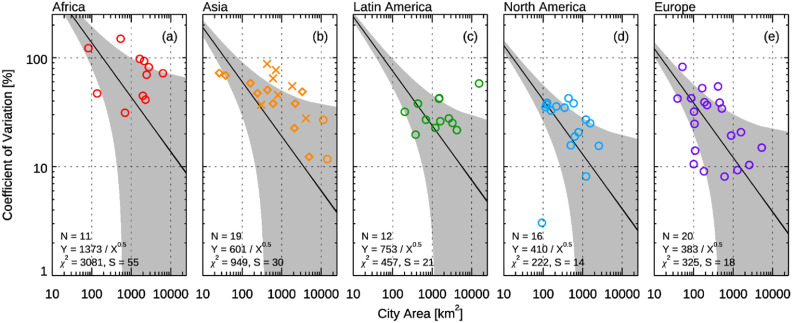
Coefficient of variation (CV) of three citywide fossil fuel CO_2_ emissions estimated from EDGAR, ODIAC, GPC inventory (CV = *σ*/*μ* × 100, where *μ* and *σ* and the mean and standard deviation of the three emission estimates) plotted as a function of cities’ geographical area. In panel (b), circle symbols are cities in Central East Asia, diamond symbols are cities in East, Southeast Asia and Oceania, and X symbols are cities in South and West Asia. Please note that the plot is on a log-log scale. Black solid lines is a non-linear regression model ($y = e/\sqrt x {\text{ }}$) fitted to available data points by minimizing the chi-squared statistic (${\chi ^2} = \mathop \sum \limits_i {\left( {{y_i} - y_i^{{\text{fitted}}}} \right)^2}/D,{\text{ where }}D{\text{ is degrees of freedom}})$. The gray area indicates the standard error of regression $\left(s = \sqrt {\mathop \sum \limits_i {{\left( {{y_i} - y_i^{{\text{fitted}}}} \right)}^2}} /\left( {N - 1} \right),{\text{ where }}N{\text{ is the number cities}}\right)$.

As a measure of variability among three citywide FFCO_2_ emissions estimates (i.e. EDGAR, ODIAC, GPC inventory), the coefficient of variation (CV) is calculated for each of the 78 C40 cities (CV = *σ*/*μ* × 100, where *μ* and *σ* and the mean and standard deviation of the three emission estimates). Figure 8 shows that values of the CV tend to be smaller for cities with larger geographical areas (i.e. the larger number of EDGAR/ODIAC grid cells aggregated). A simple regression model ${\text{CV}} = {\text{ }}e/\sqrt {{\text{City Area}}}$ is applied to data points in each region, assuming that the grid cell-level uncertainty reduces by the square root of *N* when aggregated to citywide sum emissions. Cities in North America showed the best goodness-of-fit statistic with the chi-squared (${\chi ^2}$) value of 222, followed by cities in Europe (${\chi ^2}$ = 325). Cities in Africa showed the lowest goodness-of-fit statistic (${\chi ^2}$ = 3081). The estimated coefficient e, the CV at a single cell level (1 km^2^), was lowest for European cities (383%) and highest for African cities (1373%).

#### 3.3.3. Point emission source errors in GPC inventory, EDGAR, and ODAIC: case studies of power plants in Amman, Jordan, and New Orleans, USA

For Amman, Jordan, we found inconsistent accounting of the two gas-fired power plants—Amman East Power Plant (400 MW) and Levant Power Plant (241 MW)—which generate 8% of Jordan’s electricity demand (Nebras Power 2019). According to OpenStreetMap, these two power plants are co-located within the Amman metropolitan area boundary that is used to construct GPC inventory (coordinates: 31°53′56.6″ N 36°4′47.2″ E). We found that Amman’s GPC inventories (years 2014, 2018, and 2019) do not report any power plant emissions, with the note ‘No power generation is occurring within Amman’ (Al-Raqqad 2020). In EDGAR, the geolocations of these two power plants match the OpenStreetMap coordinates. According to EDGAR, Amman’s CO_2_ emissions increased by 12.3% yr^−1^ (1.0 MtCO_2_) between 2014 and 2018, and the power plant emissions account for two third of the increase (0.64 MtCO_2_) (figure 6(a)). In ODIAC, no notable point source emissions are found at the OpenStreetMap coordinates. Such inconsistent handling of these two power units induces significant variations in the trend estimates (EDGAR: 12.3% yr^−1^, ODIAC: 2.3%yr^−1^, GPC inventory: 2.6%yr^−1^).

The Michoud power plant in New Orleans, which accounted for one-third of citywide FFCO_2_ emissions in 2014, ceased operation in 2016 (EPA 2022a). The New Orleans’ 2017 GPC inventory spreadsheet reflects the closure of the Michoud power plant, leaving the note ‘I.4.4 not occurring in 2017 due to power plant transitions’ (Siobhan Foley 2018). According to the GPC inventory, New Orleans’ FFCO_2_ emissions declined by 39% between 2014 and 2017 (−13.1% yr^−1^), and the power sector induced a decline of 34% (figure 5). Meanwhile, the Michoud power plant emissions in EDGAR and ODIAC do not reflect the shutdown of units in 2016. The resulting emission trend estimates from EDGAR and ODIAC show much slower decreasing trends than the GPC inventory (EDGAR: −1.9% yr^−1^, ODIAC: −1.6% yr^−1^).

#### 3.3.4. EFs in GPC inventories

**Figure 9.**
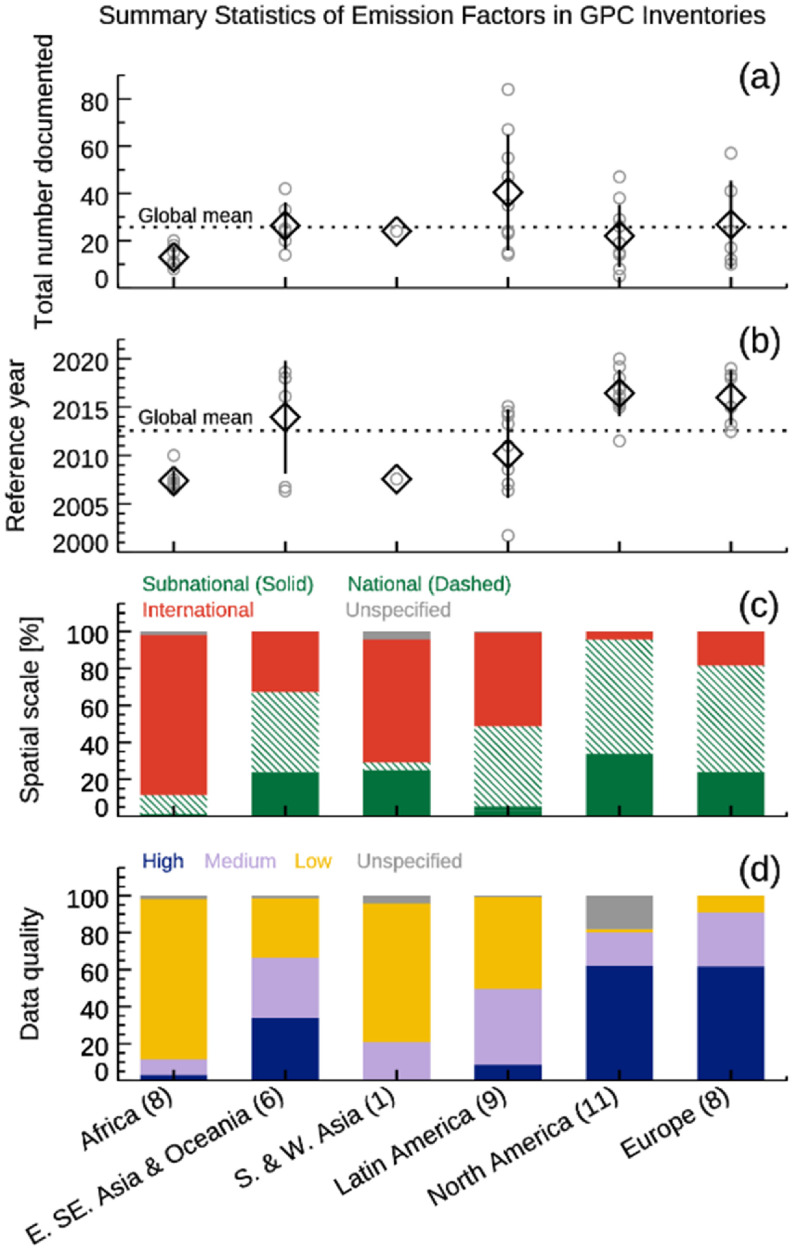
Overview of emission factors (EFs) documented in GPC inventories for 43 C40 cities in six global regions. The total number documented (a), average reference year (b), and average spatial scale of emission factors (EFs) (c) are shown. For example, emission factors from the IPCC 2006 Guideline for National Greenhouse Gas Inventories report is tagged as the reference year of 2006 and a spatial scale of ‘International’. Due to limited data availability, only one city for the S. & W. Asia region (Amman, Jordan) and none of the Central East Asian cities are included. (a), (b) The grey circle symbol indicate the citywide total in (a) and citywide mean in (b). The diamond symbol and vertical line indicate the regional mean and standard deviation. (c) Spatial scale profile of the EFs: i.e. the Solid green portion indicates the fraction of EFs labeled as ‘Local’, ‘Metro’, ‘Regional’, ‘State’, or ‘Provincial’ (subnational). (d) Data quality of the EFs: High (Navy), Medium (purple), Low (Yellow), Unspecified (Grey). (GPC guidelines recommend cities provide assessments of data quality of emission factors, following H–M–L rating).

The GPC inventories submitted through the City Inventory Reporting and Information System include a spreadsheet documenting EFs. Figure 9 shows the overview of the EFs documented for 43 cities across six regions (figure 9). Our analysis shows that North American and European cities use the most recent EFs (average reference year of 2016), and EFs are mostly subnational or national scales. International scale EFs, such as IPCC 2006 EFs, account for 4% of North American cities and 18% of European cities’ Inventory. Meanwhile, African cities use the most dated EFs (average reference year of 2007), and 87% of those EFs are international scales. Subnational scale EFs account for 1% of the EFs for African cities’ Inventory. East. Southeast Asian & Oceanian cities’ EF profiles are close to those of North American and European cities, having an average reference year of 2014 and an international EF portion of 33%. Latin American cities’ EF profile has an average reference year of 2010 and an international EF portion of 51%. Also, the GPC guideline recommends cities provide data quality assessment for EFs used for emission calculations, using a High–Medium–Low rating. European and North American cities reported the largest percentage of ‘High’ quality EFs (62%), while the African cities reported the largest percentage of ‘Low’ quality EFs (87%).

## 4. Discussions and conclusions

### 4.1. Discrepancies in citywide CO_2_ emission estimates between the two emission calculation methods: citywide emission inventory (GPC) and global gridded emissions (EDGAR and ODIAC)

For 78 C40 cities, the relative difference in annual citywide FFCO_2_ emission is −25 ± 53% between EDGAR and GPC inventory and 12 ± 62% between ODIAC and GPC inventory. Among the seven global regions, cities in Africa showed the largest variability in the relative difference (1*σ* = ±84% for EDGAR vs. GPC, 1*σ* = ±111% for ODIAC vs. GPC). This result is consistent with Chen *et al* (2020), who reported a large variability in the emission discrepancies between local inventories and ODIAC for 14 global cities, ranging from −62% (ODIAC < Inventory) for Manhattan, USA, to +148% for Cape Town, South Africa. Chen *et al* (2020) reported that the ODIAC CO_2_ emission estimates are higher than the local inventories for Cape Town (+148%), Sao Paulo (+43%), and Beijing (+40%). Our analysis shows that ODIAC’s FFCO_2_ emissions are higher than the GPC inventories for Cape Town (+91%) and Sao Paulo (+31%). Meanwhile, EDGAR’s estimates are lower than the GPC inventories for Cape Town (−25%) and Sao Paulo (−60%).

For 39 C40 cities, the difference of trend estimates is 0.5 ± 4.7% yr^−1^ for EDGAR vs. GPC and −0.3 ± 3.9% yr^−1^ for ODIAC vs. GPC. The 1 sigma values of ±4.7% yr^−1^ (EDGAR vs. GPC) and ±3.9% yr^−1^ (ODIAC vs. GPC) are greater than the rates of emission reduction that many C40 cities have pledged: i.e. net zero by 2050 from the baseline year of 2010 (−2.5% yr^−1^). This finding suggests that emission estimates from global gridded emission datasets must be significantly improved for city-level policy applications, such as emission mitigation progress tracking. The following sections discuss three primary sources of uncertainty—spatial disaggregation, point emission sources, and EFs.

### 4.2. Sources of emission discrepancies: spatial resolutions of EDGAR (0.1°)

ODIAC has been frequently used in urban CO_2_ emission studies given its 1 km spatial resolution (Janardanan *et al* 2016, Lauvaux *et al* 2016, Han *et al* 2020c). Meanwhile, EDGAR’s 0.1° resolution has been considered more suitable for regional/country scale analysis (Gately *et al* 2013, Han *et al* 2020a, Crippa *et al* 2022). Figure 7 shows that gridding 1 km pixel of ODIAC into 0.1° grid cells results in decreases in citywide emissions for 67 out of 78 C40 cities, while 11 cities show increase in emissions. When 1 km grid cells are aggregated into 0.1° grid cells, emission gradients smooth out throughout the 0.1° pixel. Such a smoothing effect can lead to either overestimation or underestimation in the citywide emissions depending on the spatial gradient of emissions surrounding the city boundary. For most cities, emissions tend to increase as the pixel gets closer to the urban center. In such cases, gridding to a coarser resolution results in underestimations in citywide emissions, as shown for Tel Aviv, Israel (figures 7(d) and (e)). For Tel Aviv, Israel, the west of the city is mostly the Mediterranean sea, with only marine emissions, resulting in the largest underestimation among 78 cities (−63%). When the 1 km pixels outside the city have larger emissions than those within the city, gridding leads to overestimating citywide emissions. For Boston, USA, several power plants are located outside of the city boundary (i.e. Kendall Power Plant and MIT Central Utility Plant, both located in Cambridge, MA), contributing to the most significant overestimation in citywide emissions among 78 cities (+183%) (figures 7(b) and (c)).

Figure 7 also shows that the emission smoothing effect tends to decrease with larger area cities, as the fraction of pixels that overlap with city boundaries decreases for larger cities. Our analysis suggests that the coarse spatial resolution of EDGAR has less of an impact on cities larger than 3000 km^2^ (Mean RD = −0.7%). For the cities between 1000 km^2^ and 3000 km^2^, relatively small underestimations are expected (−2.6%) when using EDGAR. For the cities smaller than 1000 km^2^, we recommend careful investigation of emission spatial gradients before using EDGAR, as significant bias can be introduced (−63% (Tel Aviv) ∼ +183% (Boston)).

### 4.3. Sources of emission discrepancies: spatial disaggregation error in the EDGAR and ODIAC

Andres *et al* (2016) reported an uncertainty of 120% (2*σ*) for CDIAC’s FFCO_2_ emitting grid cell over the annual timescale, and Oda *et al* (2019) reported that the emission uncertainty for a 1 km grid cell decreases by 80% when aggregated at 100 km. Our study shows that the CV for three FFCO_2_ emission estimates (i.e. EDGAR, ODIAC, and GPC inventory) tend to decrease with the increasing geographical area of cities. When a regression model of ${\text{CV}} = {\text{ }}e/\sqrt {{\text{City Area}}}$ is applied, North American cities showed the lowest standard error (*s* = 14), and African cities showed the highest standard error (*s* = 55). The regional variation in the standard error is induced by regional biases in (a) spatial proxies used for disaggregating national emissions (i.e. population settlements for EDGAR and nighttime light intensity for ODIAC), (b) point emission source error, (c) EFs used in GPC inventory, and (d) uncertainty in national FFCO_2_ emissions: Andres *et al* (2012) reported that uncertainty in national FFCO_2_ emissions ranges from a few percent (i.e. 3%–5% for the U.S.) to ∼50% for countries with poorly maintained statistical infrastructures of energy data.

A relatively higher standard error for a given region implies that the spatial disaggregation error has a relatively small impact on the region, and the other sources of uncertainty—national emissions, point sources, and EFs—have large contributions to the variability in emission estimates. We discuss the impact of discrepancies in EFs and point sources in the following sections.

### 4.4. Sources of emission discrepancies: point emission sources

Two cities—New Orleans, USA, and Amman, Jordan—show the largest discrepancies in the emission trend estimates. The case study of Amman, Jordan, shows inconsistent accounting of power plants between GPC inventory, EDGAR, and ODIAC lead to large variability in the trend estimates (EDGAR: 12.3% yr^−1^, ODIAC: 2.3% yr^−1^, GPC inventory: 2.6% yr^−1^). The case study of New Orleans, USA, reveals that EDGAR and ODIAC have some point sources that are not keeping up with the up-to-date operation status, inducing significant error in the trend estimates (GPC inventory: −13.1% yr^−1^, EDGAR: −1.9% yr^−1^, ODIAC: −1.6% yr^−1^).

For the U.S. power sector, the U.S. EPA Clean Air Markets Program Data (CAMPD) provides operation status, emissions, and geolocations of individual power-generating facilities (EPA 2022b). The CAMPD dataset enables a thorough evaluation of uncertainty in the U.S. region’s point source emission in gridded emission datasets and local inventories (Ahn *et al* 2020, Liu *et al* 2020). Currently, such a detailed power plant database is not available on a global scale. The Carbon Monitoring for Action (CARMA) is the only global database that gathers CO_2_ emissions for ∼50 000 individual power plants but has no longer been active since 2012 (Wheeler and Ummel 2008). Continuous efforts to develop a global database of power plant emissions would be essential for accurately tracking urban GHG emission mitigations for global cities.

### 4.5. Sources of emission discrepancies: emissions factors used in GPC inventories

The average reference years of EFs documented in GPC inventories are 2007 for African cities, 2010 for Latin American cities, and 2016 for North American and European cities. International scale EFs, such as the ones from IPCC 2006, account for 87% of entire EFs documented in African cities’ GPC inventory, while that only account for 4% in North American cities’ inventories. The availability of local-specific/up-to-date EFs across global regions correlates with the emissions comparison results: the African cities show the largest standard error for the CV regression (*s* = 55), while North American (*s* = 14) and European cities (*s* = 18) show the lowest standard error.

The lack of local-specific and up-to-date EFs data has been considered a major challenge in developing the GHG emissions inventory (Satterthwaite 2007, Pitt and Randolph 2009, Ibarra-Espinosa and Ynoue 2017, Bai *et al* 2018, Nagendra *et al* 2018). Baltar de Souza Leão *et al* (2020) analyzed GHG inventories for 24 Brazilian cities and found that 17 inventories possess incomplete activity data for major sectors. Arioli *et al* (2020) reviewed 73 journal articles that report city-level GHG inventory and found that most cities lack local-specific transportation data. Li *et al* (2017) adopted the GPC to estimate GHG emissions in Beijing, China, and reported that the missing data was the major challenge in developing the GPC inventory. Driscoll *et al* (2015) conducted the gap analysis of GHG emission inventories for Trondheim, Norway: they reported that the most pressing need is local-specific data, such as local transportation activity data. Our findings suggest that promoting and supporting global south cities to develop and adopt local-specific and up-to-date EFs should be prioritized to reduce discrepancies in citywide emission estimates.

### 4.6. Remote sensing of atmospheric CO_2_

Emerging satellite observations of atmospheric CO_2_, such as NASA Orbiting Carbon Observatory 3 (OCO-3), can provide independent citywide emission estimates (Nassar *et al* 2017, Fu *et al* 2019, Goldberg *et al* 2019, Andrade *et al* 2022, Bell *et al* 2020, Liu *et al* 2020, Ye *et al* 2020, Hakkarainen *et al* 2021, Kiel *et al* 2021, Park *et al* 2021, Chevallier *et al* 2022, MacDonald *et al* 2022). The major sources of uncertainties in the satellite-based approach are atmospheric transport (wind) and isolating urban enhancement signals from the background level, which is more challenging for the growing season due to active biospheric uptake (Wu *et al* 2022). With further improvements in methodologies, satellite-based estimates could bridge gaps between city inventories and gridded datasets. We suggest the following ten cities—Rotterdam, Yokohama, Buenos Aires, Kuala Lumpur, New York City, Amsterdam, Seoul, Paris, Boston, and Addis Ababa—as testbed sites for developing satellite-based emission estimate methods given (a) high per-area CO_2_ emissions (>30 ktCO_2_ km^−2^, figure 3) and (b) periodic emissions reporting from the GPC method. Improvements in the three emission tracking approaches—local emission inventories, gridded emission datasets, and atmospheric observations—would complement each method and accelerate progress toward accurate, consistent, and transparent emissions tracking.

### 4.7. Implications for stakeholders of urban climate action planning

The GPC provides detailed and consistent guidelines for cities to construct GHG emission inventories. According to GPC inventories of 78 global C40 cities, on average, scope 1 FFCO_2_ emissions account for 69% of total GHG emissions.

Our study shows that ODIAC could be used to provide first-order estimates for citywide annual FFCO_2_ emissions, especially for cities lacking reliable data-collecting systems. For 78 global cities’ annual FFCO_2_ emissions, ODIAC agrees to GPC inventory within ±62% (1*σ*). The level of agreement between ODIAC and GPC inventory varies by region: African cities show the largest variability between ODIAC and GPC. ODIAC (1 km resolution) and EDGAR (0.1° resolution) tend to show better agreement toward GPC inventory for larger cities. We recommend using EDGAR for cities larger than 1000 km^2^. For emission trend estimates, our study shows that neither the current version of ODIAC2020b nor EDGARv60 are suitable for tracking citywide emission mitigation progress at a policy-relevant scale.

The data quality of EFs used in GPC inventories varies by region, with the highest quality for European and North American and the lowest for African and Latin American cities. Updating local-specific and up-to-date EFs, which would require investments in city-scale data-collecting systems, will narrow the discrepancies in emission estimates. We also acknowledge that emerging satellite remote sensing data (i.e. NASA OCO-3) could be utilized to bridge gaps between various emission estimation methods.

## Data Availability

No new data were created or analysed in this study.
